# Sleep Apnea Events Recognition Based on Polysomnographic Recordings: A Large-Scale Multi-Channel Machine Learning approach

**DOI:** 10.1109/OJEMB.2024.3508477

**Published:** 2024-11-28

**Authors:** Nicolò La Porta, Stefano Scafa, Michela Papandrea, Filippo Molinari, Alessandro Puiatti

**Affiliations:** Faculty of InformaticsUniversità della Svizzera Italiana (USI) 27216 6900 Lugano Switzerland; Institute of Information Systems and Networking (ISIN)University of Applies Sciences and Arts of Southern Switzerland (SUPSI) 30446 6962 Lugano-Viganello Switzerland; Institute of Digital Technologies for Personalised Healthcare (MeDiTech)University of Applies Sciences and Arts of Southern Switzerland (SUPSI) 30446 6962 Lugano-Viganello Switzerland; Institute of Digital Technologies for Personalised Healthcare (MeDiTech)University of Applies Sciences and Arts of Southern Switzerland (SUPSI) 30446 6962 Lugano-Viganello Switzerland; Department of Clinical NeurosciencesLausanne University Hospital (CHUV) and University of Lausanne (UNIL) 27213 1011 Lausanne Switzerland; NeuroRestore, Defitech Centre for Interventional NeurotherapiesCHUV, UNIL, and Ecole Polytechnique Fédérale de Lausanne (EPFL) 27218 1011 Lausanne Switzerland; Institute of Information Systems and Networking (ISIN)University of Applies Sciences and Arts of Southern Switzerland (SUPSI) 30446 6962 Lugano-Viganello Switzerland; Department of Electronics and TelecommunicationsPolitecnico di Torino 19032 10129 Turin Italy; Institute of Digital Technologies for Personalised Healthcare (MeDiTech)University of Applies Sciences and Arts of Southern Switzerland (SUPSI) 30446 6962 Lugano-Viganello Switzerland

**Keywords:** Sleep-related breathing disorders, sleep apnea, obstructive sleep apnea, machine learning, polysomnography

## Abstract

*Goal:* The gold standard for detecting the presence of apneic events is a time and effort-consuming manual evaluation of type I polysomnographic recordings by experts, often not error-free. Such acquisition protocol requires dedicated facilities resulting in high costs and long waiting lists. The usage of artificial intelligence models assists the clinician's evaluation overcoming the aforementioned limitations and increasing healthcare quality. *Methods:* The present work proposes a machine learning-based approach for automatically recognizing apneic events in subjects affected by sleep apnea-hypopnea syndrome. It embraces a vast and diverse pool of subjects, the Wisconsin Sleep Cohort (WSC) database. *Results:* An overall accuracy of 87.2$\pm$1.8% is reached for the event detection task, significantly higher than other works in literature performed over the same dataset. The distinction between different types of apnea was also studied, obtaining an overall accuracy of 62.9$\pm$4.1%. *Conclusions:* The proposed approach for sleep apnea events recognition, validated over a wide pool of subjects, enlarges the landscape of possibilities for sleep apnea events recognition, identifying a subset of signals that improves State-of-the-art performance and guarantees simple interpretation.

**Abbreviations**
SAHS: Sleep Apnea-Hypopnea Syndrome
SBD: Sleep-related Breathing Disorder
CSA: Central Sleep Apnea
OSA: Obstructive Sleep Apnea
MSA: Mixed Sleep Apnea
HYP: Hypopnea
CHD: Coronary Heart Disease
HF: Heart Failure
PSG: Polysomnography
WSC: Wisconsin Sleep Cohort
AASM: American Academy of Sleep Medicine
AHI: Apnea-Hypopnea Index
GHS: Grass Heritage System
MANOVA: Multivariate Analysis Of Variance
PCA: Principal Component Analysis
PC: Principal Component
EEG: ElectroEncephaloGraphic signal
EOG: ElectroOculoGraphic signal
ECG: ElectroCardioGraphic signal
EMG: ElectroMyoGraphic signal
ICSD: International classification of sleep disorders
RIP: Resistance Inductive Plethysmography
CPAP: Continuous Positive Airway Pressure
ML: Machine Learning
TRS: Training Set
TS: Test Set
SVM: Support Vector Machine
kNN: k-Nearest Neighbour
SENS: Sensitivity
SPEC: Specificity
PPV: Positive Predictive Value
NPV: Negative Predictive Value
ACC: Accuracy

## I. Introduction

Sleep has a crucial importance in every physiological condition of the body. It relaxes the muscles, allows cell turnover and tissue regeneration and especially strengthens the central nervous system since during sleep the maximum of neuronal plasticity is reached. According to the International classification of sleep disorders (ICSD), sleep disorders were divided into eight categories: insomnias, sleep-related breathing disorders, hypersomnias of central origin not due to a circadian rhythm sleep disorder, circadian rhythm sleep disorders, parasomnias, isolated symptoms (apparent normal variants, and unresolved issues), and other sleep disorders.

### A. Sleep Apnea-Hypopnea Syndrome

Sleep Apnea-Hypopnea Syndrome (SAHS) falls within the Sleep-related Breathing Disorder (SBD) spectrum. Patients affected by SAHS experience numerous involuntary respiratory pauses during the night referred to as “apneic events”, which must last between 10 seconds to 2 minutes (typically around 20 to 40 seconds [1]) to be considered clinically significant. The duration of apneic events is influenced by several factors such as gender, obesity, age, sleep position, pharyngeal collapsibility, loop gain, with many of these factors interacting with each other [2], [3]. The airflow reduction causes a proportional drop in arterial blood oxygen saturation level, triggering an autonomic response that commonly evolves in neurophysiological awakening, disturbing the subject's rest [4]. This condition translates into different symptoms both during sleep and during the wake. Typical symptoms during sleep are loud snoring, choking sounds, and sudden body movements, while typical symptoms during wake are daytime sleepiness, fatigue, and memory-related problems. Sleep apnea is categorized into three forms:

• Central Sleep Apnea (CSA), characterized by the absence of respiratory effort due to central nervous system dysfunctions.
• Obstructive Sleep Apnea (OSA), characterized by respiratory effort hampered by the collapse of upper airway soft tissues and tongue.
• Mixed Sleep Apnea (MSA), a combination of OSA and CSA.

Respectively the 0.4%, 84%, 15% of cases in the U.S. and Europe [5]. Hypopnea (HYP), instead, is a less severe condition not pathologically comparable to apnea and continues to be an area of considerable controversy [6]. The gold standard for SAHS recognition is Type I PolySomnoGraphy (PSG) manual evaluation, which comprehends evaluating a whole night on the basis of at least seven of the following physiological parameters (signal): EEG (C4-A1 or C3-A2), EOG, EMG (chin), ECG, Airflow, Respiratory effort, and Oxygen saturation, as well as the tracks of body position and eventual leg movements. Each recording session is carried out in ad-hoc facilities within hospitals or sleep centers and require the continuous presence of specialize personnel during the whole night, resulting in high costs associated with the diagnosis of SAHS. From a technical standpoint, the evaluation procedure is very time-consuming and requires high effort from clinicians; despite being highly standardized by American Academy of Sleep Medicine (AASM) guidelines [7], it is not error-free. These limits, along with the saturation of sleep units, result in costly procedures associated with the treatment of the patients [8]. From a clinical standpoint, the impact of SAHS on the quality of life of the patients is non-negligible, and many works investigated this aspect. Subjects who suffer from SAHS have a higher probability of having cardiac and cerebral infarcts or high arterial blood pressure, as well as arrhythmias and other dysfunctions of the cardiorespiratory system [9]. In [10] the association of objectively measured SBD with incident coronary heart disease (CHD) or heart failure (HF) was studied, unrevealing an increasing trend in estimated hazard ratios with increasing SBD severity, reaching a 2.6 times more likely incidence of CHD or HF in patients with severe SBD compared to those without sleep-disordered breathing. Moreover, both total and cancer mortality show an increasing linear trend with increasing SBD severity as well as with an increasing hypoxemia index [11]. From the treatment-delivery standpoint, a huge bottleneck is represented by unawareness, with a great part of the patients being unaware of their own symptoms. AASM estimates about 29 million U.S. adults that suffer from moderate to severe OSA, with an estimated 80% living unaware of it and undiagnosed. It is obvious to understand why SAHS is a public health and economic challenge. [4]. Thus, the necessity of early identification of SAHS events for a more effective outcome of patients' treatment is crucial.

Although various devices have been used to measure physiological signals, detect apneic events, and help treat sleep apnea, significant opportunities remain to improve the quality, efficiency, and affordability of sleep apnea care.

American Academy of Sleep Medicine (AASM) digital task force identifies five basic tasks a system used to diagnose and detect breathing-related events must embrace [12]:

1) The system must allow to acquire and record data;
2) The system must allow to visualize the aforementioned data;
3) The system must allow data manipulation so that clinicians can visually assign a score to events;
4) The system must allow for data reduction. In particular, the final goal is to obtain useful diagnostic summary statistics for reporting starting from epochs;
5) The system must allow the storage of relevant data and results.

There does not exist a uniform standard for the upper-listed processes. Stages 3 and 4 are the most interesting ones in the light of the present work. A schematic view of the sleep diagnostic device types according to AASM can be found in Appendix A.

From this perspective, this paper aims to answer two research questions (RQ1 and RQ2). Firstly, if it is possible to describe the sleep apnea-related SBD condition of the patients detecting apneic events exploiting only low-invasive-to-record signals from PSG. This research question is of primary importance in the perspective of obtaining simpler and less cumbersome diagnostic devices. In particular, we aim to obtain a system capable of accomplishing the task, which uses the minimum number of parameters (signals) to be considered a type-3 sleep diagnostic device (see Table IV in Appendix A). Secondly, if it is possible to perform such description based on machine learning methods that do not extend to ensembles or deep learning solutions, thus excluding completely black-box approaches. This stringent constraint aims to reduce clinicians' doubts about what the model do to return predictions. Several works, such as [14], [15], [16] suggest how fundamental is to build by design a system with a good tradeoff between accuracy and interpretability, thus increasing the thrustworthiness of the system from the clinicians standpoint. Moreover, ML models have the benefit of being light-weight models with respect to ensembles or deep learning approaches, resulting in a suitable choice for the implementation into portable devices (RQ1).

We aim to address RQ1 and RQ2 by exploiting a large and variegated dataset, surpassing the size of those used in comparable studies. In this way, the system will take great advantage of the dataset heterogeneity, increasing the models' generalizability over new unseen data. The present work's novelty relies on the identification of an apnea detection system's optimal configuration in terms of both features and models, which ensures a good tradeoff between system simplicity, model interpretability, and discriminative power by design. Such configuration can be employed as an algorithmic backbone for type-3 sleep diagnostic devices (see Appendix A), given that the models examined are lightweight enough to be implemented in portable systems yet powerful enough to correctly accomplish the detection task.

The rest of the paper is organized as follows. Section II presents the proposed approach describing the data used and their processing, the features extracted, and the choice of the ML models. The results are presented from five different points of view, depending on the clusters considered and the incisiveness of the dimensionality reduction applied, in Section III and are discussed in Section IV. Finally, a conclusion is made in Section V.

**TABLE I:** Subjects Demographic, Clinical, and PSG-Related Characteristics of WSC Database

	**Male (n=848)**	**Female (n=739)**
Age (years)	58 $\pm$ 8	56 $\pm$ 8
BMI (kg/m^2^)	31 $\pm$ 6	33 $\pm$ 8
Neck circumference (cm)	41 $\pm$ 3	36 $\pm$ 4
TST* (h)	5.9 $\pm$ 0.9	6.4 $\pm$ 0.9
CPAP (n users)	51	33

* TST: Total Sleep Time, measured as the total time spent in sleep stages N1, N2, N3, and REM.

## II. Method

### A. Database and Signals Description

In the present work, we used the Wisconsin Sleep Cohort (WSC) database [17], [18], which comes from an ongoing longitudinal study that started more than 20 years ago. This dataset includes subjects with and without cardiovascular disease, CPAP users, subjects already suffering from SAHS of any severity classified with the apnea-hypopnea index (AHI), and subjects who have never received a diagnosis (see Table I for subjects details). It comprises 2570 PSGs gathered with two different acquisition systems, 1800 with the Grass Heritage System (GHS) and 770 with the Grass Comet Lab Based system. To be consistent, we used data collected only from GHS since most of the data were collected with it. From the total of 1800 records, we removed the ones presenting missing data or very noisy signals, ending with 1587 records. The PSGs collected with the Grass Heritage System include the following signals: 2 EEG, 2 EOG, 2 EMG, 1 ECG, 1 audio registration, nasal- and oral airflow, 1 nasal pressure, 3 RIP-belt volume signals (thoracic, abdominal, and sum), 1 body position, and 1 blood saturation (SpO$_{2}$). All the signals within the dataset were sampled at 100 Hz with a 16-bit resolution ADC and were already analogically pre-filtered with a pass band-filter to remove the stationary component and frequencies above 30 Hz. The WSC database comprehends both the raw data and the true labels of apneic events. These labels were manually assigned by experts according to the scoring procedure reported in Appendix C.

To meet RQ1, in our study, we excluded the most invasive-to-record signals, namely nasal- and oral airflow and nasal pressure, usually collected through cannulas. Studies like [21] and [22] highlight how the usage of cannulas can cause discomfort in patients. Moreover, we considered only the thoracic signals among the three RIP-belt volume signals.

### B. Signal Processing and Feature Extraction

To be consistent with the AASM guidelines [7] each signal was segmented into non-overlapping 30-second epochs, discarding the ones affected by sensor detachments and the epochs of wakefulness. The features extracted can be grouped into three main categories: time-based statistics, describing the timeseries distribution, complexity, quantifying the presence of long-range correlations in non-stationary time series, and frequency-based. Moreover, according to the nature of the signals, some signal-specific features were extracted, e.g. hypoxic burden features for SpO$_{2}$, RR intervals-based features for ECG, position encoding, etc.

Finally, a dataset of 973’000 epochs described by 130 variables was obtained.

### C. Case Studies and Dimensionality Reduction

**Fig. 1.**
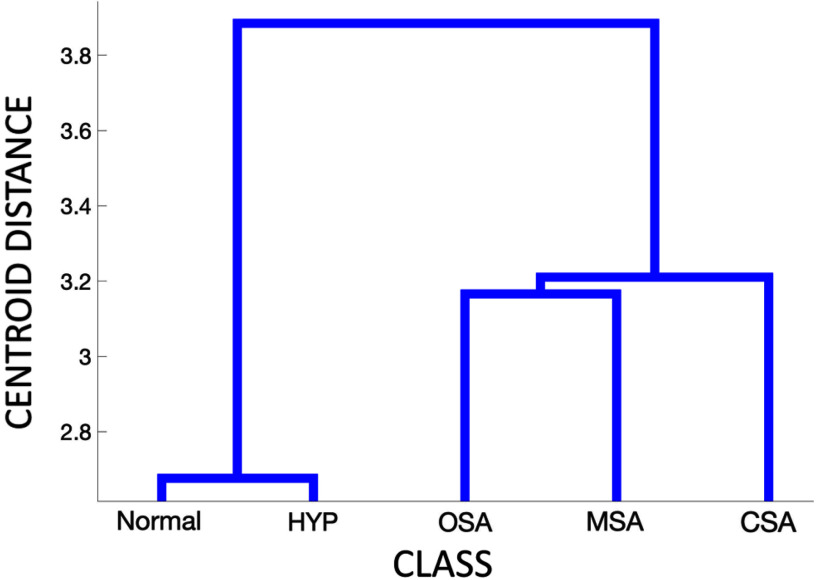
MANOVA dendrogram considering all 5 classes.

To better understand how the available dataset's variance can help discriminate the different classes, firstly a Multivariate ANalysis Of VAriance (MANOVA) [30], [31] was performed. Fig. 1 shows a dendrogram plot of the group means obtained from the MANOVA that displays two main clusters: one containing normal and hypopnea epochs and one containing apnea epochs (OSA, CSA, and MSA).

In this work we analyzed the following dichotomous classification tasks:

i) Apnea vs Normal/Hypopnea
ii) Apnea vs Normal
iii) OSA/MSA vs. CSA

**TABLE II:** 32-Feature Subset

**THORACIC VOLUME (n = 16)**	**BLOOD SATURATION (n = 9)**
TIME-BASED [25]	TIME-BASED [23]
Mean, std dev, median, IQR	Std dev, range, min
number of peaks, min PH	95%ile, 5%ile, $\Delta$ I
mean PH, std dev PH	HYPOXIC BURDEN
sum of PH, mean IPD, AUC	TSA95
FREQUENCY-BASED [25]	COMPLEXITY [24]
Mean, median, and peak	ApEn025, ApEn015
frequencies, band power	
COMPLEXITY	
Renyi's entropy	
**AUDIO (n = 4)**	**EOG (n = 3)**
TIME-BASED [25]	FREQUENCY-BASED [27]
Mean, median	Delta rhythm power of
FREQUENCY-BASED [26]	right and left EOGs,
ISE range and time	beta rhythm power
spent outside the range,	of right EOG
mean(ISE)$\pm$2*std(ISE)	

PH = Peaks Height, IPD = Inter-Peak Distance, $\Delta$ I = Delta Index, ISE = Instantaneous Spectral Entropy

**TABLE III:** 44-Feature Subset

**ECG (n = 18)**	**THORACIC VOLUME (n = 12)**
FREQUENCY-BASED [28]	TIME-BASED [25]
IQRs of 3rd, 4th and 5th	Std dev, IQR, number of peaks,
detail levels of DWT decomposition,	min PH, mean PH, std dev PH,
variance of 1st, 2nd and 3d	sum oh PH, mean IPD,
detail levels of DWT decomposition,	skewness IPD, AUC
standard deviation and MAD of	FREQUENCY-BASED [25]
all detail levels of DWT decomposition	Mean frequency, band power
RR INTERVAL-BASED [29]	COMPLEXITY
Mean and median of RR intervals	Renyi's entropy
**BLOOD SATURATION (n = 9)**	**AUDIO (n = 4)**
TIME-BASED [25]	FREQUENCY-BASED [26]
Std dev, range, minimum,	Energy in 3rd and 4th detail
M2, 5%ile, $\Delta$ I	level of DWT decomposition
HYPOXIC BURDEN [23]	COMPLEXITY
TSA95	ISE range
COMPLEXITY [24]	Renyi's entropy
ApEn025, ApEn015	
**BODY POSITION (n=1)**	
Numerical encoding	

Cases (i) and (ii) reflect the main objective of the work (RQ1), being apnea detection tasks. However, we decided to investigate also case (iii), in order to further classify the type of apnea. Cases i and ii aim to differentiate the apnea condition from other condition, while case iii aims to distinguish between different types of apnea. Secondly, a correlation analysis was performed highlighting a correlation among features derived from the same signals. Therefore, the dataset underwent a feature dimensionality reduction process. In particular, for the apnea detection cases (i and ii), Principal Component Analysis (PCA) [32] was performed over the covariance matrix of the initial dataset after features z-score normalization. Then, a parallel coordinate chart was plotted to check the amount of variance explained by a limited number of Principal Components (PCs) and retain only the features that weighed most within those PCs. Two subsets were obtained from this process: (a) 78-feature-subset and (b) 32-feature-subset. The former preserved features from most of the signals and was obtained through a looser selection, while the latter was obtained through a more stringent approach, discarding EEG, ECG, and position features (see Table II).

The same approach was then applied for the apnea distinction, and a 44-feature-subset was obtained, including 9 SpO$_{2}$, 18 ECG, 1 position, 4 audio, and 12 thoracic volume features (see Table III). It is noteworthy that 18 of the 33 ECG features were retained, whereas in previous cases, they were discarded completely.

**Fig. 2.**
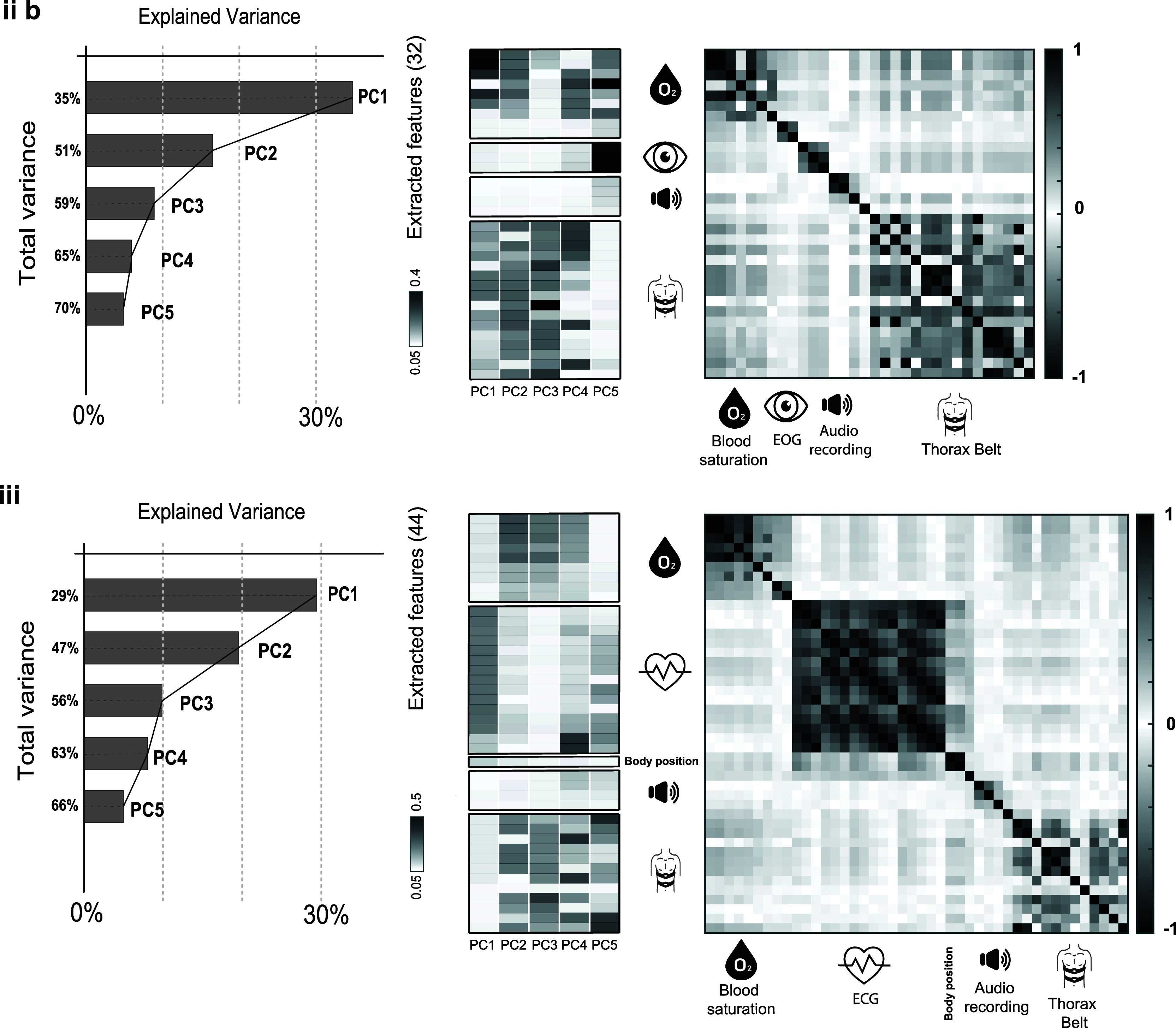
Principal components and correlation analyses for case studies iib (upper row) and iii (lower row). The leftmost graphs represent the cumulative variance charts; the central graphs represent heatmaps of the first five PCs stratified per biosignal; the rightmost graphs represent the correlation matrices.

**Fig. 3.**
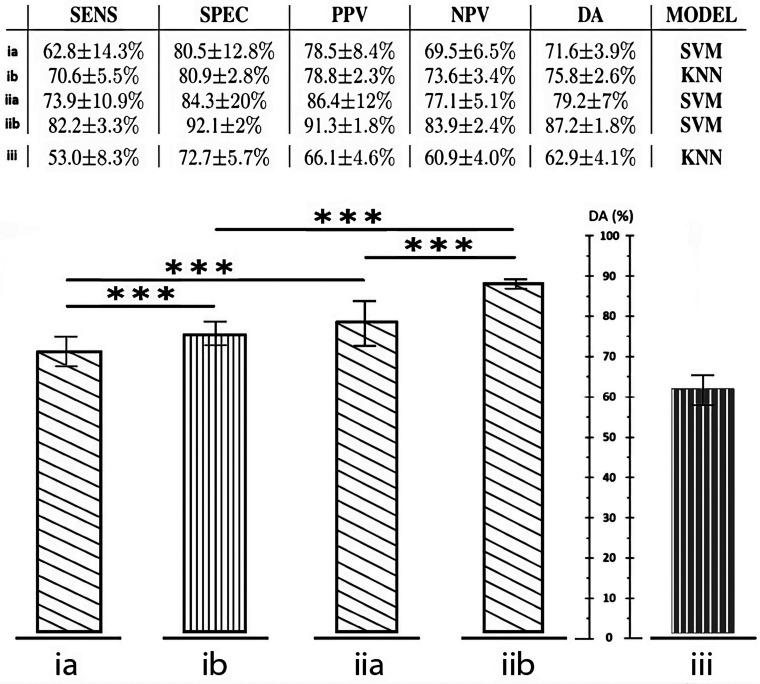
Comparison of Diagnostic Accuracy (DA) for the best model of each case study on TS (*** = p-value $< $ 0.001). The table in the top-right corner contains the prediction metrics under the form mean $\pm$ standard deviation. **(ia)** Norm/Hyp vs Apn (78 features). **(ib)** Norm/Hyp vs Apn (32 features). **(iia)** Norm vs Apn (78 features). **(iib)** Norm vs Apn (32 features). **(iii)** OSA/MSA vs CSA (44 features).

Fig. 2 shows insights about PCA and correlation analysis. The upper part refers to case study (iib) while the lower part refers to case study (iii), normal breathing vs apnea and OSA/MSA vs CSA respectively. The leftmost graphs are the Pareto charts or explained variance charts, obtained through PCA analysis. They show how much each Principal Component (PC) contributes to explain the variance (information) contained in the dataset. The horizontal axis refers to the explained variance while the vertical axis refers to the cumulative variance, so, for instance, in case iib the first PC (PC1) contributes to explaining 35% of the total variance, while the second PC (PC2) contributes to explaining the 16% of the total variance that summed up with PC1 reaches 51%, and so on. The central graphs represent heatmaps of the principal components stratified according to biosignals. The color code of these heatmaps goes from lighter colors to darker colors based on the weights a certain feature has within a certain principal component: the darker the color, the more weight a certain feature brings to the PC. For instance, in case (iib) the blood-saturation-derived features weigh more than the audio features. Ultimately, the rightmost graphs represent the correlation matrices calculated over the subset of features that characterize each case study. These matrices can be seen as a composition of blocks belonging to different domains, the different biosignals, as it is possible to infer from the icons along the two axes. It is clear how there is a certain amount of residual correlation among features derived from the same signals while there is almost no correlation among features derived from different signals.

### D. Training, Test, and Model Fitting

The datasets obtained after feature selections were divided into a training set (TRS) and a test set (TS). Both datasets were balanced in terms of the number of events (normal, hypopnea, OSA, CSA, and MSA). Since the datasets were composed of a variable number of epochs per subject, to avoid overfitting, they were firstly divided into TRS (70%) and TS (30%), randomizing on subjects, ensuring that a subject present in the TRS was not present also in the TS. Then different case-wise balancing approaches were applied: in both cases (i) and (ii), TRS and TS were balanced by taking all the apnea epochs and randomly picking the same amount of normal-hypopnea or normal epochs. In case (iii), instead, we kept all the MSA epochs, which are the less represented, and we randomly selected OSA and CSA epochs, maintaining the apnea events proportion with respect to the MSA.

### E. Model Choice and Tuning

In order to meet RQ2, five different supervised learning algorithms have been applied to predict the apnea events: Decision Trees, Discriminant Analysis, Naïve Bayes, Support Vector Machine (SVM), and k-Nearest Neighbour (kNN).

The best tradeoff was chosen between training speed, memory usage, and interoperability. The metrics chosen for comparison are Sensitivity (SENS), Specificity (SPEC), Positive Predictive Value (PPV), Negative Predictive Value (NPV), and Accuracy (ACC) [19] [20].

The equations for metrics computation are reported below.

\begin{align*}
{\mathbf{Sensitivity}} &= \frac{\text{True Positives}}{\text{True Positives} + \text{False Negatives}}\\
{\mathbf{Specificity}} &= \frac{\text{True Negatives}}{\text{True Negatives} + \text{False Positives}}\\
{\mathbf{PPV}} &= \frac{\text{True Positives}}{\text{True Positives} + \text{False Positives}}\\
{\mathbf{NPV}} &= \frac{\text{True Negatives}}{\text{True Negatives} + \text{False Negatives}}\\
{\mathbf{Accuracy}} &= \frac{\text{True Positives} + \text{True Negatives}}{\text{Total Population}}
\end{align*}

All models were trained with the same TRS, and the hyperparameters were optimized according to the built-in routines present in MATLAB which leverage on bayesian optimization [33]. Moreover, to obtain a better estimate of the predictive accuracy, a 5-fold cross-validation was performed.

## III. Results

The classification performance of all the models were calculated over the same TS for a fair comparison. The number of iterations per model was fixed to 200. The ground truth for this work is the manual evaluation of the same PSGs by experts. For each case study the best result in terms of tradeoff between diagnostic accuracy and balanced sensitivity and specificity is reported and the resulting prediction metrics are reported in Fig. 3 under the form mean $\pm$ standard deviation. In section IV the letter *a* will indicate the 78-feature-dataset, while the letter *b* will indicate the 32-feature-dataset.

## IV. Discussion

Focusing on the apnea detection task (case (i) and case (ii)), SVM models proved to be the best tradeoff in terms of performance, and the most informative features resulted from SpO$_{2}$ and thoracic volume signals. Moreover, after the hyperparameter optimization, all the SVM models were tuned using a linear kernel (for a schematic view of the main hyperparameters obtained during the optimization process see Appendix B for a more detailed description). The outcome of the feature selection, especially for case (ii), suggests the feasibility of implementing a simple acquisition system suitable for a home setting that only entails using a pulse oximeter and RIP bands. The performance of the models over the apnea distinction task were suboptimal because the same dataset of the apnea detection task was utilized despite being a separate classification problem. It is evident how searching for more specific features for this task will boost the models' performance (a starting point could be investigating the ECG features, which emerged as the most informative in this case). This suboptimal choice of the dataset for this task translated into the non-unique choice of the distance metric after the optimization process (see Appendix B). In general, taking into account HYP in cases (i) and (ii), and MSA in case (iii) deteriorates model performance since these intermediate conditions cause overlapping between data distributions in the respective case studies. Lastly, the more radical feature selection improved performance in terms of both mean and standard deviations of all prediction metrics in both cases (i) and (ii) in favor of a reduced number of collectible signals. The present work positively answers to both the research questions: the subset of this condition can be the basis for cheaper, less-cumbersome and easier-to-use type 3 sleep diagnostic device, which can leverage on the proposed apnea detection approach. Such systems will be more compliant for the patient and by design more trustworthy for the clinician.

The novel approach presented in this work also demonstrates how simple ML models can perform well over a variegated dataset containing many subjects. The literature shows a broad spectrum of approaches for both apnea detection and apnea distinction tasks. Reviews such as [34], [35], [36], and [37] can simplify the comparison with our method and help make qualitative considerations by examining other single- or multi-channel ML approaches using open-access databases (neither neural networks nor ensemble classifiers are taken into account). However, there is a limited number of studies carried out over a vast number of patients, and even fewer utilize the same database. In particular, only two out of more than one hundred studies reviewed in the aforementioned works allow a fair comparison. In particular, [34] reports a study with SENS 93.1$\%$ and PPV 97% on a pool of only 10 subjects, very few compared to the 1587 subjects used in the present work. [35], instead, reports another study on 1479 subjects with SENS and PPV of only 68.60$\%$ and 66.36% respectively, which are considerably lower than our results.

The current work is limited by several factors. Firstly, the non-negligible inter- and intra-operator variability [38]. Despite the scoring procedure is highly standardized, the database has been collected over more than 20 years by different experts. Secondly, the hardware prefiltering of the signals, which translated into a loss of spectral information for some of them, such as EEG and EOG. Thirdly, the results highlighted that the thoracic belt respitrace is one of the most informative signals. According to the WSC manual of operation [40], these signals were collected through semi-disposable RIP belts, which have been shown to produce less reliable output with respect to disposable cut-to-fit and disposable snap-on RIP belts [39]. From a technical standpoint, ensemble classifiers and neural network-based approaches could be more effective in solving the tasks of the presented work. However, it was decided to investigate standard ML approaches in order to meet RQ2. Finally, despite having analyzed a variegated pool of subjects, the population considered for the current work was still limited, and further inclusion of a different pool of subjects would have helped for a better generalization.

## V. Conclusion

The present study proposed a novel ML-based approach for automated detection and distinction of apneic events starting from conventional PSG data. Different ML models and combination of features have been examinated in order to identify the optimal configuration. Both the research questions have been addressed; it has been demonstrated how the usage of low-invasive-to-record signals is feasible for the detection of apneic events. The performed analyses evidenced how blood saturation and respitrace signals are the most informative for the detection task, while the ECG is the most informative for the distinction task. Moreover, it has been demonstrated that standard ML approaches are powerful enough to solve the apneic detection task. Further, as discussed in Section IV, studies could build upon the current work by improving feature selection and hyperparameter optimization processes to explore the potentialities of this dataset. Furthermore, other studies could focus on extracting more informative features for specific classification tasks retaining only the most discriminant ones identified in the current work (e.g. a more in-depth analysis of the distinction task based on ECG, SpO$_{2}$, and/or respitrace signals). Finally, more fine-grained detection approaches are currently under our investigation. The intention is to craft a cascade system where the current approach is used to identify apneic epochs while a further approach is used to identify the exact extension of apneic events within these epochs. This would considerably help experts' evaluation because knowing the exact number and duration of the apneic event can give a deeper insight into the pathological condition of the patients in terms of the severity of SAHS measured through AHI.

*Authors contributions:* Conceptualization, N.L.P., F.M. and P.A.; Methodology, N.L.P. and S.S.; Software, N.L.P.; Validation, N.L.P. and S.S.; Formal analysis, N.L.P. and S.S.; Investigation, N.L.P.; Resources, N.L.P. and P.A.; Data curation, N.L.P.; Writing—original draft preparation, N.L.P. and S.S.; Writing—review and editing, N.L.P. and S.S.; Visualization, N.L.P. and S.S.; Supervision, M.P., F.M and P.A.; Project administration, N.L.P. and P.A.; Funding acquisition, NONE. All authors have read and agreed to the published version of the manuscript.

*Conflicts of interest:* The authors declare no conflicts of interest in the current study.

*Informed consent:* Not applicable.

*Funding:* This research received no external funding.

## Appendix A

This appendix contains a schematic view of the sleep diagnostic device types according to AASM. For each type are indicated the possible parameters, the possible parameters (physiological signals) are indicated, as well as other important characteristics.

**TABLE IV:** Sleep Diagnostic Devices Types According to AASM [13]

	**TYPE 1^1^**	**TYPE 2^2^**	**TYPE 3^3^**	**TYPE 4^4^**
Parameters	At least seven: - EEG (C4-A1 or C3-A2) - EOG - EMG (chin) - ECG - Airflow - Respiratory effort - Oxygen saturation	At least seven: - EEG (C4-A1 or C3-A2) - EOG - EMG (chin) - ECG or heart rate - Airflow - Respiratory effort - Oxygen saturation	At least four: - Ventilation (at least two channels of respiratory movement, or respiratory movement and airflow) - ECG or heart rate - Oxygen saturation	At least one
Body position	Documented	Not mandatory	Not mandatory	Not mandatory
Leg movement<	EMG or motion sensor	EMG or motion sensor	Not mandatory	Not mandatory
Personnel	Always present	Not present	Not present	Not present
Intervention	Possible	Not possible	Not possible	Not possible

*Type 1:* Standard PSG system. *Type 2:* Comprehensive portable PSG.

*Type 3:* Modified portable sleep apnea testing. *Type 4:* Continuous single- or dual-bioparameter recording.

## Appendix B

This appendix contains a brief summary of the main hyperparameters obtained from the optimization process. For a more schematic view, they were collected in Table V. The notation of values between brackets is: (median, 25%ile, 75%ile).

**TABLE V:** Main Hyperparameters

**Case Study [Model]**	**Main Hyperparameters**
ia [SVM]	Kernel function: linear
Kernel Scale: (211.85; 76.89; 536.80)	
Box Constraint: (0.09; 0.01; 1.12)	
ib [kNN]	Distance: standardize euclidean
num. neighbours: (11; 9; 13)	
iia [SVM]	Kernel function: linear
Kernel Scale: (47.46; 18.63; 234.86)	
Box Constraint: (0.05; 0.01; 1.08)	
iib [SVM]	Kernel function: linear
Kernel Scale: (95.50; 1.75; 382.00)	
Box Constraint: (12.17; 0.01; 387.61)	
iii [kNN]	Distance: standardize euclidean (50%)
mahalanobis (50%)	
num. neighbours: (5; 3; 7)	

It is noteworthy how the optimization process led the SVM models to use linear kernels. From a technical standpoint, this is a great advantage because this kernel choice speed up the computations with respect to other nonlinear kernels.

Further, the kNN models were tuned over a reasonable number of neighbours considering the numerosity of the dataset. Furthermore, the kNN model for apnea distinction was tuned half of the time over a standardized euclidean distance and half of the time over a mahalanobis distance. The latter is a distance metric that takes into account the data distribution (it can measure how far away a data point is from the distribution, while Euclidean distance can not), so this means that the distribution of the datapoints plays a key role in this case which differs from the other cases.

## Appendix C

In this appendix will be deepened the definitions and the scoring procedures of apneas and hypopnea according to the WSC manual of operations [40] followed by clinicians to score the breathing-related events of the database exploited for this work.

### A. Apneas

*Definitions:* Apneas are characterized by no indication of airflow in nasal pressure, no detectable breathing pattern in the thermistor and a clear amplitude reduction in effort, followed by an associated desaturation. The different types of apnea have been distinguished observing the thermocouple and the respitrace signals: **OSA** (no indication of airflow by thermocouple and an indication of effort in respitrace channels), **CSA** (no indication of airflow by thermocouple and no indication of effort in respitrace channels), and **MSA** (no indication of airflow by thermocouple and areas of no effort followed by effort in respitrace channels).

*Scoring Procedure*

1) Determine if there is flow or no flow. Criteria for NO flow:
  • Does not follow the previous pattern of flow **and/or** is $< 20\%$ of amplitude of the largest previous breath (determined by $\mu V/mm$ of unclipped airflow sensitivity, if necessary) **and** has an interruption of airflow that is $>10$ seconds in duration.
2) Determine if there is effort or no effort. Criteria for no-effort (from Respitrace):
  • Does not follow the previous pattern of breathing **and** has no discernable amplitude of the signal in the respitrace.
3) Measure duration of event:
  • Measure from the beginning of the last expiration on the airflow channels to the beginning of the next inspiration on the airflow channels to determine the 10-second criterion;
  • If 10 seconds, measure the duration of the event from the beginning of the last expiration to the beginning of the next inspiration on the SUM channel (sum of volumes) of the respitrace that best corresponds to the points of measurement of duration in the airflow channels;
  • If not 10 seconds, determine if the event meets the criterion for a hypopnea: 4% desaturation. If it does not, then the event is ignored and not scored;
  • NOTE: When the event is obviously an apnea and is between 9.5 and 10 seconds, round the duration up to 10 seconds and score.

### B. Hypopnea

*Definition:* A discernable decrease in flow in the nasal pressure channel and/or thermistor with an associated oxygen desaturation of 4% or greater indicated in the SpO$_{2}$ channel beginning in sleep.

*Scoring Procedure*

1) Use a display view of at least a 120-second window;
2) Determine a discernable decrease in the SUM channel (sum of volumes) defined as a $>50\%$ decrease in the mean amplitude of the three largest breaths preceding the onset of the event, or a clear reduction in amplitude that is $< 50\%$ with an associated oxygen desaturation of $>4\%$;
3) Measure the duration of the event:
  • Measure from the beginning of the last expiration on the SUM to the beginning of the next inspiration on the SUM to determine the 10-second criterion (from the beginning to the end of the event);
  • If not 10 seconds, delete the event mark;
  • Mark the desaturation event on the SpO$_{2}$ channel following the respiratory event, beginning within 30 seconds of the end of the respiratory event;
  • Delete the desaturation event for a hypopnea if the desaturation is $< 4\%$;
  • Determine that the desaturation occurs in sleep. Events that begin in sleep and end in wake are always scored. Events that begin and end in the wake are never scored;
  • Mark the beginning and end of the event in the SpO$_{2}$ channel corresponding to the desaturation. Duration of desaturation events should not be greater than 120sec.
4) Mark the corresponding event in the SUM channel as Hypopnea if associated with a desaturation of $> 4\%$;
5) Without the presence of an adequate signal in the nasal pressure channel, use the nasal/oral thermistor channel for determination of flow.
